# Using tDCS to Explore the Role of the Right Temporo-Parietal Junction in Theory of Mind and Cognitive Empathy

**DOI:** 10.3389/fpsyg.2016.00380

**Published:** 2016-03-15

**Authors:** Xiaoqin Mai, Wenli Zhang, Xinmu Hu, Zhen Zhen, Zhenhua Xu, Jing Zhang, Chao Liu

**Affiliations:** ^1^Department of Psychology, Renmin University of ChinaBeijing, China; ^2^State Key Laboratory of Cognitive Neuroscience and Learning, Beijing Normal UniversityBeijing, China

**Keywords:** social cognition, theory of mind, cognitive empathy, temporo-parietal junction (TPJ), transcranial direct current stimulation (tDCS)

## Abstract

The right temporo-parietal junction (rTPJ) is thought to be closely related to theory of mind (ToM) and cognitive empathy. In the present study, we investigated whether these socio-cognitive abilities could be modulated with non-invasive transcranial direct current stimulation (tDCS) of the rTPJ. Participants received anodal (excitatory), cathodal (inhibitory), or sham stimulation before performing a social cognitive task which included inferring other’s intention (the ToM condition) and inferring other’s emotion (the cognitive empathy condition). Our results showed that the accuracy of both ToM and cognitive empathy decreased after receiving the cathodal stimulation, suggesting that altering the cortical excitability in the rTPJ could influence human’s socio-cognitive abilities. The results of this study emphasize the critical role of the rTPJ in ToM and cognitive empathy and demonstrate that these socio-cognitive abilities could be modulated by the tDCS.

## Introduction

Successful human social interaction mostly depends on the understanding of others’ mental states, which are built on the social abilities such as theory of mind (ToM) and cognitive empathy. ToM is the central function of human social cognition, referring to the ability to attribute other’s mental states, such as beliefs and intentions (Frith and Frith, 1999). It can help us understand observable actions by inferring agents’ mental representations. Affective empathy is the ability to share the emotional experience of others (“I feel what you feel”). It is different from cognitive empathy which is the capacity to understand other’s perspective or mental states (Preston and de Waal, 2002; Shamay-Tsoory et al., 2009; Schnell et al., 2011). Cognitive empathy emphasizes inferring other’s affective mental state (“I understand what you feel”) but not necessarily sharing this feeling (de Waal, 2008). Many researchers believe that cognitive empathy is related to ToM (Eslinger, 1998; Shamay-Tsoory et al., 2003). Walter (2012) proposes that cognitive empathy is the emotional part of ToM. Blair (2005) even equates cognitive empathy with ToM. Since ToM and cognitive empathy have an overlap in concept, they might recruit common brain regions.

Functional magnetic resonance imaging (fMRI) studies have demonstrated the involvement of temporo-parietal junction (TPJ) in the attribution of mental states (Vogeley et al., 2001; Saxe and Kanwisher, 2003; Saxe and Wexler, 2005; Spengler et al., 2009; Schurz and Perner, 2015). For example, Saxe and Kanwisher (2003) used false belief tasks and found the greater activity in the TPJ when participants read stories about a person’s belief than the stories describing a physical process, such as melting or rusting, suggesting the important role of the TPJ in understanding other’s mental states. Some studies also suggests that the TPJ is involved in cognitive empathy (Rankin et al., 2005; Shamay-Tsoory et al., 2005; Vollm et al., 2006). Vollm et al. (2006) designed an experimental paradigm to directly compare the neural correlates of ToM and cognitive empathy using fMRI, in which participants were asked to make inferences about the emotional or the intentional states of the protagonists in a visual cartoon task. The results showed that both ToM and cognitive empathy activated the TPJ, medial prefrontal cortex, and temporal poles, though they also recruited other distinct brain regions. They concluded that the overlapping network of ToM and cognitive empathy is related to inferring others’ internal states. Using the same cartoon task, Atique et al. (2011) also found that both ToM and cognitive empathy (in their paper, they use the term “intention mentalizing” and “emotion mentalizing”, respectively) activated the TPJ, though different subregions. These findings suggest that ToM and cognitive empathy are closely related and the TPJ is engaged in both of them.

In recent years, non-invasive brain stimulation techniques, such as transcranial magnetic stimulation (TMS) and transcranial direct current stimulation (tDCS), have been used to explore the role of the TPJ, especially the right TPJ (rTPJ), in social cognition. Costa et al. (2008) reported that the application of repetitive TMS over the rTPJ significantly worsened accuracy and response times (RTs) in both false belief and faux-pas written story tasks, confirming the important role of the rTPJ in ToM performance. Some studies have also found that applying the TMS over the rTPJ disrupted the capacity to use mental states in moral decision making (Young et al., 2010; Jeurissen et al., 2014). Moreover, Young et al. (2010) revealed that using TMS over the rTPJ led participants rely less on the actor’s mental states when making moral judgments.

More recently, using tDCS, several studies reported that the application of anodal or cathodal stimulation over the rTPJ could modulate the belief attribution (Sellaro et al., 2015; Sowden et al., 2015; Ye et al., 2015). Sellaro et al. (2015) found that participants who received anodal stimulation assigned less blame to accidental harms compared to participants who received cathodal or sham stimulation, emphasizing the role of rTPJ in mediating the belief attribution for moral judgements. Ye et al. (2015) reported that inhibiting the rTPJ of typical adults with cathodal stimulation decreased the role of beliefs in moral judgments and increased the dependence on the action’s outcomes when making moral judgment.

However, Santiesteban et al. (2012) did not find the effects of anodal or cathodal stimulation of the rTPJ when participants were asked to make mental judgments about themselves or others (ToM), though they did observe that anodal stimulation of the rTPJ improved the on-line control of self and other representations in the imitation and perspective-taking tasks which involve low-level sociocognitive processes. In their latest study (Santiesteban et al., 2015), they applied anodal stimulation over the rTPJ or left TPJ (lTPJ) and observed the bilateral TPJ involvement in the perspective-taking and imitation inhibition, while they still found no effect on the ToM task in which participants watched a movie and were asked to infer the mental state of characters. The possible reason for no tDCS effects on ToM in their studies might be that the ToM tasks they used are insensitive to the performance variation induced by stimulation in typical adults (Santiesteban et al., 2015).

The present study aimed to use the non-invasive tDCS to examine whether both ToM and cognitive empathy depends causally on the neural activity in the rTPJ that has previously identified through fMRI (Vollm et al., 2006; Atique et al., 2011). Therefore, in the present study, we used the cartoon task derived from Vollm et al. (2006) to validate the crucial role of rTPJ in the these sociocognitive abilities through elevating or inhibiting the cortical excitability. Many fMRI studies of ToM reported the activity in the rTPJ or bilateral TPJ, while a few studies reported the unilateral activation of the left TPJ (see meta-analysis by Van Overwalle, 2009). In addition, most previous TMS and tDCS studies of ToM stimulate the rTPJ (e.g., Costa et al., 2008; Santiesteban et al., 2012). In order to make our study comparable to previous studies, we focused on the rTPJ instead of lTPJ. Based on the previous studies, we hypothesized that anodal stimulation could enhance the abilities of ToM and cognitive empathy, while the cathodal could have the opposite effect. As to our knowledge, this is the first study to explore whether both ToM and cognitive empathy could be modulated by the tDCS of the rTPJ.

There are several reasons for why we are interested in ToM in adults. First, although the ToM ability is already fully developed at the age of 4–5 years, its specific neural mechanisms remain unclear. Second, the ToM ability is closely correlated with neurodevelopmental disorders, such as autism. Third, some socio-cognitive abilities, such as moral judgment, and lie detection, also rely on ToM (Sellaro et al., 2015; Sowden et al., 2015; Ye et al., 2015; Young et al., 2010). Therefore, investigating ToM in adults could help us understand not only ToM itself but also neural models of other socio-cognitive abilities, as well as help individuals with neurodevelopmental disorders.

## Materials and Methods

### Participants

Sixty-eight right-handed adults (mean age 22.8 ± 2.6 years, 35 females) participated in the study. They were randomly assigned to three groups: the anodal (*n* = 21), the cathodal (*n* = 23), or the control “sham” (*n* = 24). Two additional participants, one in the anodal group and another in the cathodal group, withdrew from the study because they reported to be afraid of receiving the tDCS. None of the participants reported a history of neurological or psychiatric disorders. All participants were paid for their participation and gave their informed consent. The study was approved by the Institutional Review Board of Department of Psychology at Renmin University of China.

### tDCS Protocol

**FIGURE 1:**
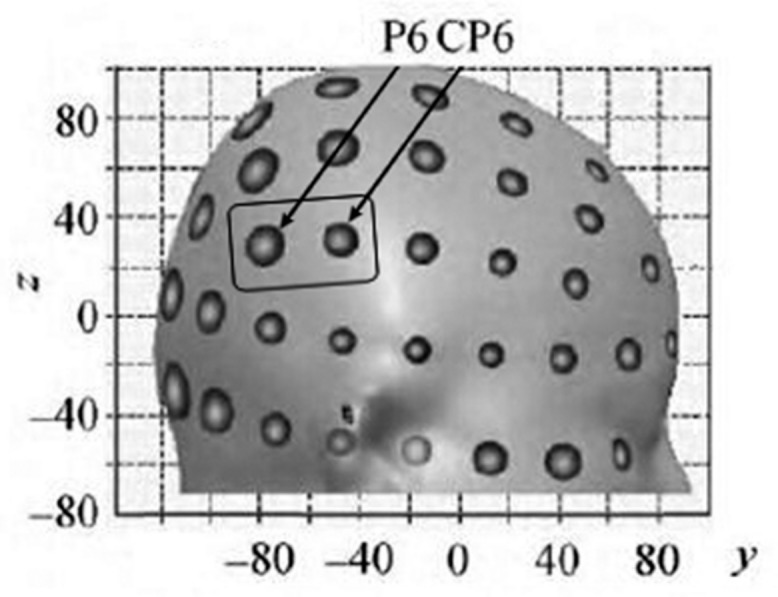
**The location of the anodal or cathodal electrode was placed in the MNI coordinates (adapted from Jurcak et al., 2007)**.

Transcranial direct current stimulation was administered through a specially battery-driven constant current stimulator (DC-Stimulator Plus, NeuroConn GmbH, Germany). The stimulation was induced through a saline-soaked pair of surface sponge electrodes (35 cm^2^ in size). To stimulate the rTPJ, the anodal or cathodal electrode was placed between CP6 and C6 according to the international 10–20 EEG system and previous fMRI studies (Jurcak et al., 2007) (see **Figure 1**). This area covers the MNI coordinates [54, -59, 22] of the rTPJ reported in previous fMRI studies (Young et al., 2007; Young and Saxe, 2009). The reference electrode was placed over the left cheek.

As in previous studies, to assure the target cortex to be activated completely (Jurcak et al., 2007; Cerruti and Schlaug, 2009), a relatively weak current (1.5 mA) was constantly delivered for 20 min. For the sham group, although the electrode was placed over the rTPJ for 20 min, the stimulation only lasted for 15 s. At the onset of each condition (anodal, cathodal, or sham), the fade in and fade out time were both 15 s (Cerruti and Schlaug, 2009; Holland et al., 2011; Keeser et al., 2011). Participants felt the current as itching sensation at the beginning of the stimulation.

### Experimental Procedure

All participants filled in the Interpersonal Reactivity Index (IRI) questionnaire (Davis, 1980) to assess empathy before receiving the stimulation. The IRI is a self-report questionnaire that includes four subscales, perspective- taking, fantasy, empathic concern, and personal distress. Each subscale is comprised of seven questions, which constitutes the total 28 items. The perspective- taking and fantasy subscales are two cognitive scales, while the empathic concern and personal distress subscales are two affective scales (Davis, 1983). We focused our analysis on the perspective- taking and fantasy subscales, because we only interested in cognitive empathy. The perspective- taking scale measures the tendency to accept spontaneously others’ point of view (e.g., “I sometimes try to understand my friends better by imagining how things looks from their perspective”); while the fantasy scale measures the tendency to transfer oneself into fictional situations (e.g., “When I am reading an interesting story or novel I imagine how I would feel if the events in the story were happening to me”). Responses are on a 5-point Likert scale ranging from 0 (does not describe me well) to 4 (describe me very well). The Cronbach’s alpha was 0.81 for the perspective-taking scale and 0.79 for the fantasy scales.

**FIGURE 2:**
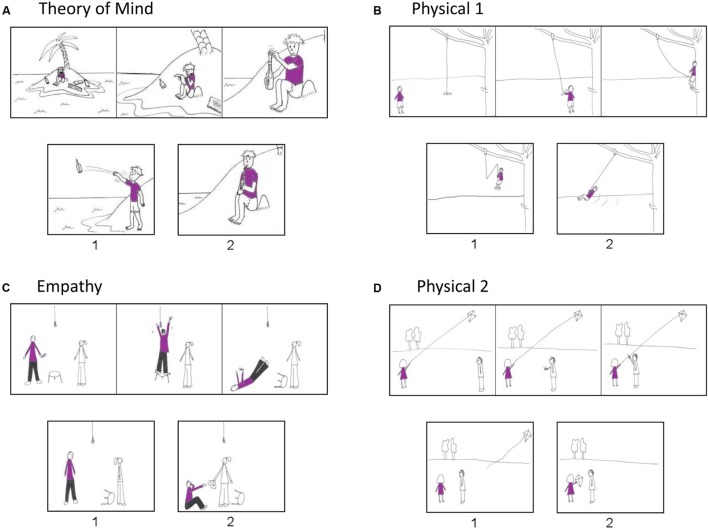
**Examples of stimuli from four conditions.** The correct answers in **(A)** Theory of mind (ToM), **(B)** Physical 1, **(C)** Empathy, and **(D)** Physical 2 stories are picture 1, picture 2, picture 2, and picture 1, respectively (derived from Vollm et al., 2006).

After the stimulation, participant performed the task derived from Vollm et al. (2006) on the computer. The task consists of four conditions: ToM, Cognitive empathy, Physical 1, and Physical 2. The Physical 1 and Physical 2 are the control of the ToM and Cognitive empathy, respectively. **Figure 2** shows examples of stimuli from each condition. In the ToM and Cognitive empathy conditions, participants were asked to infer the character’s intention or emotion, respectively. In the other two conditions, they had to make inferences based on physical causalities. The stories of ToM and Physical 1 described one character only while Cognitive empathy and Physical 2 described two characters.

A total of 40 comic strips each depicting a short story were presented in eight blocks, with each block comprising of five comic strips belonging to the same condition. Thus each condition was showed twice and each strip was showed only once. The sequence of blocks and comic strips in each block were counterbalanced.

Each block began with an introductory question for 6 s which indicated the required type of inference (ToM condition: “What will the main character do next?”; Cognitive empathy condition: “What will make the main character feel better?”; Physical 1 and Physical 2 conditions: “What is most likely to happen next?”). Each strip cartoon was presented for 6 s, and then another two cartoons showing the possible outcome were imposed on the bottom of the screen for 4.5 s. Participants were required to make a choice between the two possible outcomes of the stories by pressing the button as soon as possible. Accuracy and RTs were recorded for all cartoons. A score of one referred to a correct answer while a score of 0 was assigned to be wrong.

### Data Analysis

Analyses were done with SPSS statistical software (version 22, Chicago, IL, USA). Accuracy and RTs in each condition (ToM, Cognitive empathy, Physical 1, and Physical 2) were analyzed using one-way analysis of variance (ANOVA) with Group as a between-subjects factor (i.e., anodal, sham, and cathodal). Further *Post hoc* analyses were conducted using the Tukey’s honestly significant difference (HSD) test when appropriate.

## Results

### IRI score

The scores for the perspective-taking and fantasy subscales which measure cognitive empathy were analyzed using a one-way ANOVA with Group as a between-subjects factor (i.e., anodal, sham, and cathodal). No significant differences were found for scores of both perspective-taking (mean ± SD, 17.9 ± 3.2, 17.9 ± 3.1, and 18.8 ± 4.5) and fantasy (15.2 ± 5.0, 15.4 ± 5.8, and 14.0 ± 6.4) among the Anodal, Cathodal, and Sham groups before stimulation, indicating that there were no group differences in cognitive empathy before receiving the tDCS.

### Accuracy

**FIGURE 3:**
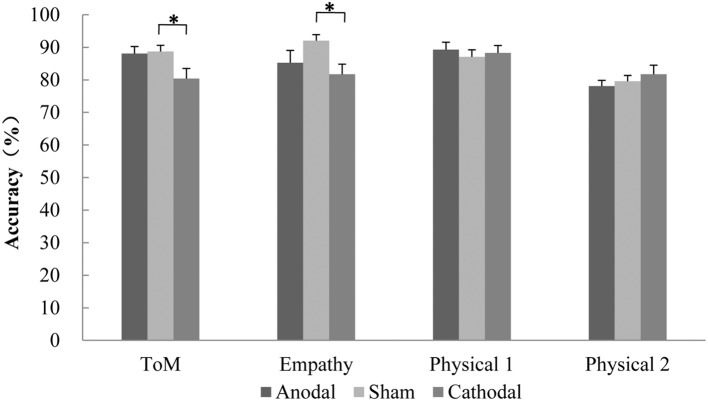
**Mean accuracy in each task condition for three stimulation groups.** Error bars indicate SEM (standard error of the mean). ^∗^*P* < 0.05.

**Figure 3** shows accuracy in four conditions for three stimulation groups. In the ToM condition, there was a reliable main effect of Group, *F*(2,65) = 3.76, *p* = 0.028. *Post hoc* analyses revealed that the difference between Cathodal and Sham groups reached significance (*p* = 0.040) but no differences were found between Anodal and Shame groups (*p* = 0.980) and between Anodal and Cathodal groups (*p* = 0.075), indicating that compared with the the Sham group, participants in the Cathodal group were less accurate in inferring the character’s intention. In the Physical 1 condition which is the control of the ToM condition, no differences were found between stimulation groups.

In the cognitive empathy condition, there was aslo a reliable main effect of Group, *F*(2,65) = 3.35, *p* = 0.041. *Post hoc* analyses showed that the difference between Cathodal and Sham groups reached significance (*p* = 0.035) but no differences were found between Anodal and Shame groups (*p* = 0.236) and between Anodal and Cathodal groups (*p* = 0.685), indicating that compared with the Sham group, participants were less accurate in inferring the character’s emotion after receiving the cathodal stimulation. In the Physical 2 conditions which is the control of the cognitive empathy condition, no differences were found between stimulation groups.

### Response Times

Response times were recorded for all 40 trials and the trials failing to respond within 4.5 s were excluded from the analsyis. **Table 1** shows RTs in four conditions for three stimulation groups. For each condition, the one-way ANOVA did not found any siginficant between-group effects, indicating that the tDCS over the rTPJ did not significantly affect RTs.

**Table 1:** Response times (ms) in each task condition for three stimulation groups (M ± SD).

	ToM	Empathy	Physical 1	Physical 2
Anodal	2161 ± 363	2197 ± 446	2162 ± 459	2286 ± 418
Sham	2098 ± 374	2178 ± 386	2180 ± 452	2375 ± 403
Cathodal	2085 ± 433	2177 ± 400	2137 ± 585	2273 ± 586


## Discussion

The present study assessed the potential effect of the tDCS of the rTPJ on ToM and cognitive empathy. Our findings indicate that the accuracy of both ToM and cognitive empathy decreased after receiving the cathodal stimulation, suggesting that altering the cortical excitability in the rTPJ could influence human’s socio-cognitive abilities. More specifically, the inhibition of cortical excitability by the tDCS in the rTPJ could impair human’s ability in inferring others’ mental states or emotional states. Therefore, the results of this study emphasize the critical role of the rTPJ in ToM and cognitive empathy.

The results of the present study are in line with the findings of previous fMRI studies in which they have found that the rTPJ is closely related to both ToM and cognitive empathy (Vogeley et al., 2001; Saxe and Kanwisher, 2003; Vollm et al., 2006; Spengler et al., 2009; Atique et al., 2011). They are also consistent with the TMS study by Costa et al. (2008) in which they reported that the application of the rTMS over the rTPJ could worsen ToM performances, as well as the tDCS study of Ye et al. (2015) in which they observed that the inhibition of the rTPJ with cathodal stimulation decreased the role of beliefs in moral judgments. Together with previous studies, our findings suggest that ToM and cognitive empathy could be related to the neural activity in the rTPJ.

However, Santiesteban et al. (2012) did not observe the inhibition effect of cathodal stimulation in the rTPJ on ToM. One likely reason of the discrepancy is that the reaction time, which is the only behavioral measure of the ToM test in their study, might not be a sensitive behavioral index of ToM performance. In our study, both accuracy and reaction times were measured in the ToM task and the cathodal-inhibition effects were shown only in accuracy data but not reaction times. In both studies of Santiesteban et al. (2012) and ours, participants’ reaction times are more than 2000 ms. Such slow reaction times might be insensitive to measuring the variation of the ToM ability. More recently, Santiesteban et al. (2015) further examined the bilateral TPJ involvement in ToM using tDCS and accuracy was measured in the ToM task. Unfortunately, they only applied anodal stimulation but did not use cathodal stimulation and thus the effect of cathodal stimulation of TPJ on ToM is unknown in their study.

In addition, we did not validate our hypothesis that anodal stimulation might enhance the performance of ToM and cognitive empathy. Santiesteban et al. (2012, 2015) did not find any reliable effect of anodal stimulation of the rTPJ on ToM either. The possible explanation is that the tasks in their studies and ours are so easy for healthy adults that their performances (accuracy and RTs) of the ToM and cognitive empathy were already good enough when without any stimulation and thus could not be improved with anodal stimulation. This interpretation should be tested in the future studies by investigating individuals with ToM impairment, such as people with autism spectrum disorders and schizophrenia.

In the present study, we used a between-subject design and found that tDCS of the rTPJ had effects on ToM and cognive empathy. It should ackownlege that our argument that stimulation makes a differece would be stronger if we use a within-subject design. However, the task we used in the present study is not appropriate for the within-subject design. For the within-subject design, there should be two tests, one before stimulation and another after stimulatoin. But there was only 10 trials in each condition in our task and there would not be enough trials for each test if we split 10 trials into two tests, i.e., pre-stimulation and after-stimulation tests. In addition, there might be learning and priming effects if the task is repeated after stimulation.

It has to be acknowledged that there are several limitations of the present study. First, the findings are based on one study in which the experimental materials and task came from the study of Vollm et al. (2006). However, some evidence shows that brain areas related to ToM differ for different tasks and stimuli (see the review by Schurz and Perner, 2015). Therefore, our results may not be generalizable to other ToM tasks with different stimuli. Moreover, the task might be too easy for healthy adults to validate the enhancement of anodal stimulation on ToM and cognitive empathy. Third, although previous studies have confirmed that the tDCS could induce excitability changes of the cortex (Jaberzadeh and Zoghi, 2013), we must admit that the tDCS does not have the spatial specificity and thus it is impossible to distinguish different functions of subdivisions of the TPJ. Also, different brain areas might be involved in the different types of ToM tasks (see the review by Schurz and Perner, 2015). Therefore, in the future studies, more tasks should be tried and the role of the different subregions of the TPJ should be explored through combining other neuroimaging techniques. Despite these limitations, the findings of this study still could help us understand the relationship between the rTPJ and some socio-cognitive abilities, such as ToM and cognitive empathy, and also highlight the potential contribution of the tDCS to the field of social cognition.

The tDCS technique is mainly used in clinical treatment and rehabilitation (Nair et al., 2011; Schlaug et al., 2011), while the results of the present study also show the significant value of this technology in the normal group. Some other socio-cognitive abilities also rely on ToM, such as moral judgment and lie detection (Sellaro et al., 2015; Sowden et al., 2015; Ye et al., 2015). Therefore, investigating the neural mechanisms of ToM in adults could help us understand not only ToM itself but also other socio-cognitive abilities. In addition, depression, anti-social personality disorder and stroke patients have been characterized by local changes in the brain structure and function (Grefkes and Fink, 2011; Palazidou, 2012) and thus most of them may have problems in understanding others’ intentions and emotion. The tDCS, as a non-invasive stimulation, has a wide application prospect in the improvement of such disabled patients. Future studies should also address how such clinical diseases can be modulated by the tDCS.

As far as we know, this is the first study to explore tDCS effects on both ToM and cognitive empathy by controlling the cerebral cortex excitability of the rTPJ. Future research can continuously investigate the anodal effect of tDCS over the rTPJ on ToM and cognitive empathy or apply tDCS over the left temporal area, the frontal area, and other brain areas related to social cognitive processing.

## Author Contributions

XM, CL, and WZ designed the study. WZ, ZZ, and ZX performed data collection and analysis. XM, WZ, XH, CL, and JZ wrote the paper. All authors approved the final version of the paper for submission.

## Conflict of Interest Statement

The authors declare that the research was conducted in the absence of any commercial or financial relationships that could be construed as a potential conflict of interest.

The reviewer SO and handling Editor declared their shared affiliation, and the handling Editor states that the process nevertheless met the standards of a fair and objective review.
